# Supplementary material for: Ingestible hydrogel device
Source: Nat Commun. 2019 Jan 30;10:493. doi: 10.1038/s41467-019-08355-2 (PMC6353937; doi:10.1038/s41467-019-08355-2)
Supplement: Supplementary file 1 — Supplementary Information [file 41467_2019_8355_MOESM1_ESM.pdf]

Supplementary Information for

Ingestible Hydrogel Device

Liu et al.

Supplementary Note 1

Analysis on the swelling ratio at equilibrium

To quantitatively analyze the swelling ratio of the hydrogel device, we develop a model that describes the swelling of polyelectrolyte networks as well as the inflation of the elastic membrane. Two primary components determine the swelling kinetics of the hydrogel device being i) superabsorbent particles (polyelectrolyte hydrogel) and ii) the elastic membrane with pores (Supplementary Figure 6a). When medium (e.g., water or gastric fluid) flows through the pores within the elastic membrane and subsequently migrates into the aggregation of superabsorbent particles, the particles swells and inflates the elastic encapsulation membrane [1,2]. The swelling-induced mechanical pressure exerted at the outer surface of the polyelectrolyte hydrogel P_{swelling} decreases as expansion ratio λ in length (or λ^3 in volume) increases. The polyelectrolyte hydrogel continuously swells until P_{swelling} equals the pressure required to inflate the elastic membrane $P_{\text{inflation}}$. To calculate the final swelling ratio λ_{eq}^3 of the hydrogel device, we will focus on the equilibrium state, in which the electrochemical potential of each mobile species is uniform throughout the polyelectrolyte hydrogels and the external solution [2,3].

To obtain the swelling-induced mechanical pressure exerted at the outer surface of the polyelectrolyte hydrogel P_{swelling}, we start from the free energy density of the polyelectrolyte hydrogel, which can be expressed as [2,3]

$$W = W_{net} + W_{sol} + W_{ion} + \Pi\left(1 + \sum v_a C_a - \det \mathbf{F}\right), \qquad (1)$$

where W_{net}, W_{sol} and W_{ion} are the contributions from stretching of the polymer network, mixing the polymers and the solvent, and mixing the solvent and ions. Π is the Lagrange multiplier that

enforces the volume conservation of all constituents. v_a is the volume of one mobile species a, C_a is the number of the mobile species a divided by the volume of the dry polymer, $v_a C_a$ is the total volume of species a divided by the volume of the dry polymer, and $\mathbf{F}$ is the deformation gradient of the polymer network. The free energy of stretching the polymer network can be expressed as

$$W_{net} = \frac{1}{2} NkT\left(F_{iK}F_{iK} - 3 - 2\log(\det \mathbf{F})\right), \tag{2}$$

where N is the number of polymer chains divided by the volume of the dry polymer, $kT = 4.11 \times 10^{-21}\,\mathrm{J}$, $\mathbf{F}$ is the deformation gradient of the polymer network and F_{iK} is the corresponding component. Specifically, for a spherical hydrogel with isotropic swelling ratio of λ, $F_{iK}F_{iK} = 3\lambda^2$ and $\det \mathbf{F} = \lambda^3$. The free energy of mixing the polymers and the solvent can be expressed as

$$W_{sol} = \frac{kT}{v_s}\left(v_s C_s \log \frac{v_s C_s}{1 + v_s C_s} - \frac{\chi}{1 + v_s C_s} \right), \tag{3}$$

where v_s is the volume of one solvent molecule (i.e., water), C_s is the number of the solvent molecules divided by the volume of the dry polymer. χ characterizes the enthalpy of mixing of the polymer chains and the solvent molecules. The free energy of mixing the ions and the solvent is taken to be

$$W_{ion} = kT \sum_i C_i \left(\log \frac{C_i}{v_s C_s c_i^0} - 1 \right), \tag{4}$$

where C_i is the number of the ionic species i divided by the volume of the dry polymer, and c_i^0 defines the reference concentration for zero chemical potential.

For solvents in the external solution, the contribution of free energy only comes from mixing solvents and ionic species; therefore, the chemical potential of the solvent in the external solution is

$$\mu_{s,external} = -kT\sum_i \frac{C_i}{C_s} . \tag{5}$$

For solvents inside the hydrogel, the free energy additionally covers the contribution from mixing of the polymers. Therefore, the chemical potential of the solvent in the hydrogel is

$$\mu_{s,hydrogel} = kT\left[\log\frac{v_s C_s}{1+v_s C_s} + \frac{1}{1+v_s C_s} + \frac{\chi}{\left(1+v_s C_s\right)^2} - \sum_i \frac{C_i}{C_s}\right] + \Pi v_s . \tag{6}$$

Here, we focus on the case that highly charged polyelectrolyte hydrogels swell in a dilute solution in which the ion concentration in the external solution is much lower than the fixed charge concentration in the hydrogel. Therefore, the chemical potential in external solution can be approximated to be zero (i.e., $\mu_{s,external} = 0$). The ionizable groups on the polymers are considered to be fully dissociated with C_0 being the number of the fixed negative charge divided by the volume of the dry polymer. Electro-neutrality requires that the number of the counter ions within the hydrogel equals the number of the fixed charges in the polymer, which is denoted by

$$C^- = 0 \, , \; C^+ = C_0 \tag{7}$$

Equating the chemical potential of the solvent inside the hydrogel to the chemical potential of the external solution (i.e., $\mu_{s,hydrogel} = \mu_{s,external}$), we obtain

$$\Pi = -\frac{kT}{v_s}\left[\log\frac{v_s C_s}{1+v_s C_s} + \frac{1}{1+v_s C_s} + \frac{\chi}{\left(1+v_s C_s\right)^2} - \frac{C_0}{C_s}\right], \tag{8}$$

On the other hand, the osmosis swelling of the hydrogel induces the mechanical stress inside the hydrogel when it is constrained at the expansion ratio of λ. The swelling-induced true stress of the hydrogel can be obtained by [1]

$$\sigma_{ij} = \left(\frac{\partial W}{\partial F_{iK}}\right)\frac{F_{jK}}{\det \mathbf{F}} - \Pi\delta_{ij}. \tag{9}$$

By substituting the deformation gradient, the radial true stress is

$$\sigma_{rr} = NkT(\lambda^{-1} - \lambda^{-3}) - \Pi, \tag{10}$$

The condition of incompressibility of the polymers and the solvents become

$$1 + v^s C^s = \lambda^3, \tag{11}$$

The swelling-induced pressure P_{swelling} (= σ_{rr}) can be obtained by combining Supplementary Equations 8-11

$$P_{\text{swelling}} = NkT(\lambda^{-1} - \lambda^{-3}) + \frac{kT}{v_s}\left[\log\left(1-\lambda^{-3}\right) + \lambda^{-3} + \chi\lambda^{-6} - \frac{v_s C_0}{\lambda^3 - 1}\right]. \tag{12}$$

In Supplementary Figure 6b, we plot the numerical results for swelling-induced pressure at different constrained expansion ratios. In this numerical calculation, we set $1/Nv = 1000$ with v being the volume of a single monomer $v = 1\times10^{-27}\,\text{m}^3$ [4], which means that each polymer chain contains 10^3 monomers; set $\chi = 0.1$, indicating slightly positive enthalpy of mixing; and assume $v_s = v$ [3]. The swelling-induced pressure increases as the constrained expansion ratio decreases. The intercept of x-axis gives the free swelling ratio λ_{free}.

We next calculate the pressure for inflation of the elastic membrane. We take the membrane as a neo-Hookean solid. The inflation pressure versus the expansion ratio is expressed as [1]

$$P_{\text{inflation}} = 0, \quad \lambda < \lambda_{crump} \tag{13}$$

$$P_{\text{inflation}} = \frac{2Gt_o}{R_0}\left[\left(\lambda / \lambda_{crump}\right)^{-1} - \left(\lambda / \lambda_{crump}\right)^{-7}\right], \quad \lambda > \lambda_{crump} \tag{14}$$

where G is shear modulus of the membrane, t_0 and R_0 are thickness and radius of the membrane, respectively, and λ_{crump} accounts for the degree of crumple. Here, we set $t_0 / R_0 = 0.1$ and $\lambda_{crump} = 1.2$. Supplementary Figure 6c plots the numerical results for inflation pressure versus expansion ratio for a set of membranes with different modulus. The intersection of the inflation curve and swelling curve determines the critical swelling ratio λ_{eq} at equilibrium state of the hydrogel device (Supplementary Figure 6d). As shown in Supplementary Figure 6e, the swelling ratio at equilibrium can be tuned by altering the modulus of the membrane. For an extremely soft membrane, the swelling ratio of the hydrogel device approaches the limit of free swelling ratio λ_{free}^3; for an extremely stiff membrane, the swelling ratio is bounded by the degree of crumple $\lambda_{crumple}^3$.

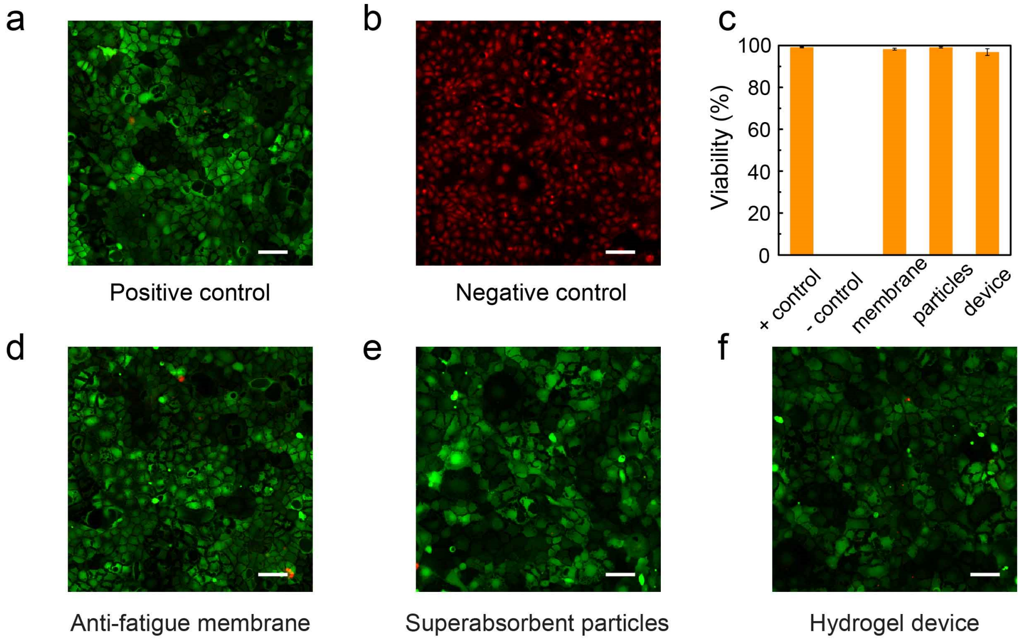

Supplementary Figure 1. Cell compatibility of the ingestible hydrogel devices. Cell viability is tested on Caco-2 cells with medium that was pre-exposed to the hydrogel device and its integral parts for one day at 37 °C. Cells without treatment and cells treated with 70% ethanol serve as positive (**a**) and negative (**b**) control, respectively. Cell viability after 72 h incubation with medium pre-exposed to the anti-fatigue membrane (**d**), superabsorbent particles (**e**), and the whole hydrogel device (**f**) are presented, respectively. Green indicates viable cells and red indicates dead cells. (**c**) The viability is calculated by the ratio of viable cells to all cells in the images, and the values represent the mean ± s.d. (N = 3). Scale bars are 100 μm.

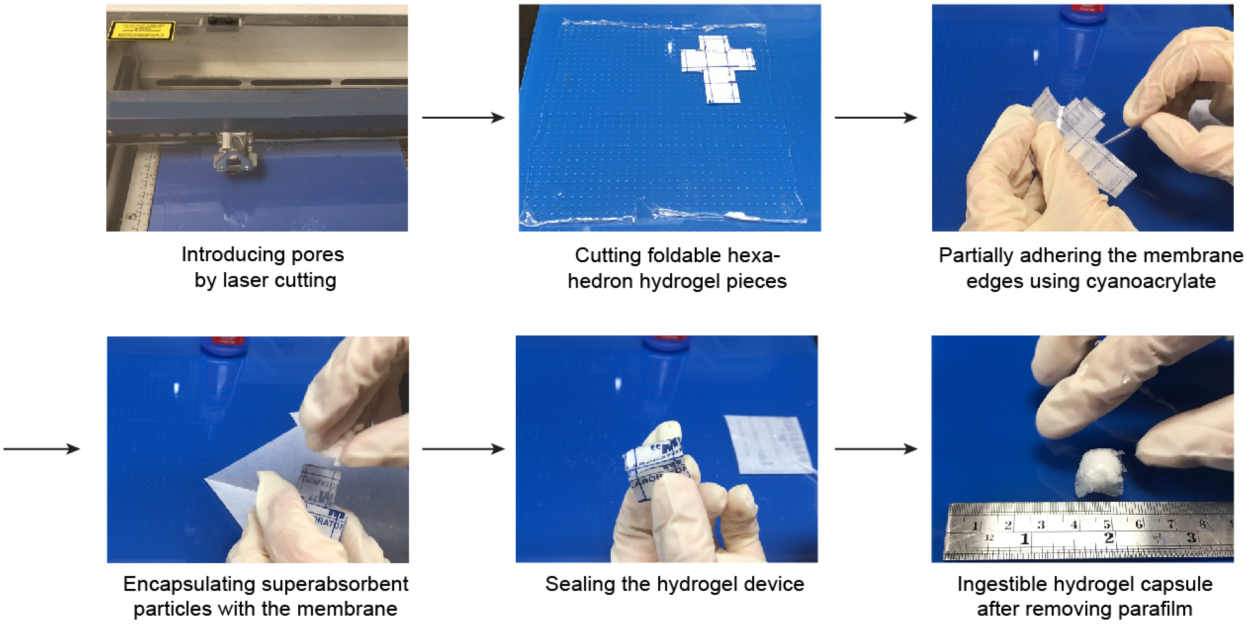

Supplementary Figure 2. Fabrication of the ingestible hydrogel devices with a cube shape. The superabsorbent particles and a porous hydrogel membrane are assembled into a hydrogel device with cube shape.

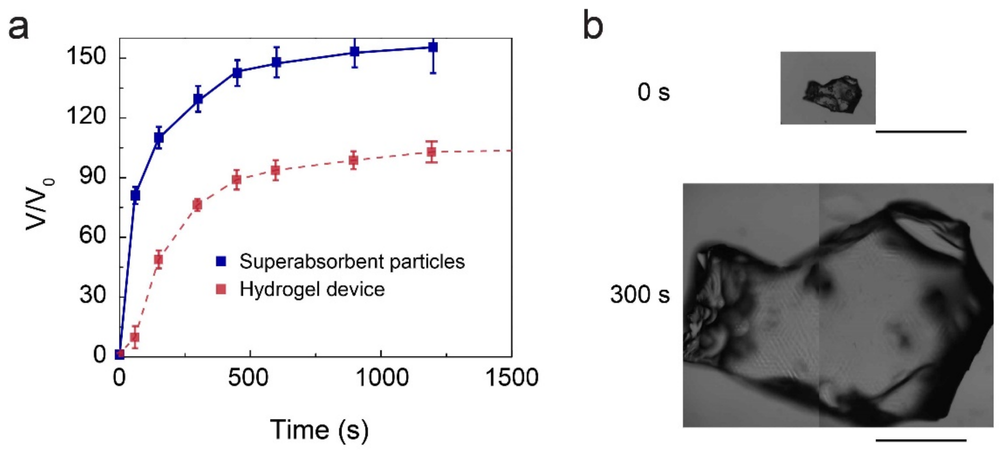

Supplementary Figure 3. Swelling of superabsorbent particles. (**a**) Volumetric swelling kinetics of superabsorbent particles without hydrogel membrane in water (blue) in comparison to the hydrogel device in water (red). Values represent the mean ± s.d. (N = 3). (**b**) Microscopic images of superabsorbent particles before and after swelling. Scale bars are 1 mm.

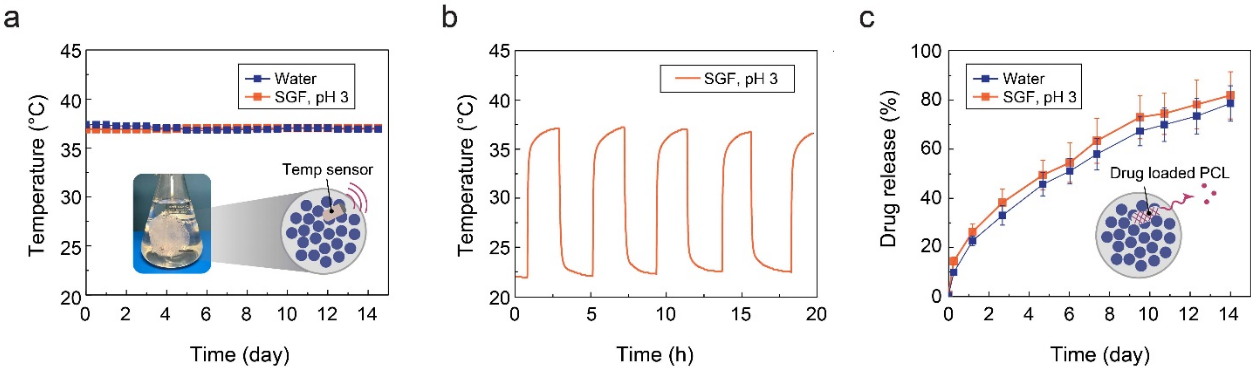

Supplementary Figure 4. ***In vitro*** **sensing and drug release capacity of the ingestible hydrogel devices.** (**a**) Temperature pattern of media placed in an incubator set to 37°C and monitored by use of an ingestible hydrogel device comprising a temperature sensor (N = 1). (**b**) Temperature pattern as described in (**a**) with alternating placement of the ingestible hydrogel device at room temperature (N = 1). (**c**) Extended caffeine release from a polycaprolyactone-based formulation incorporated in the ingestible hydrogel device at 37°C. Values represent the mean ± s.d. (N = 3).

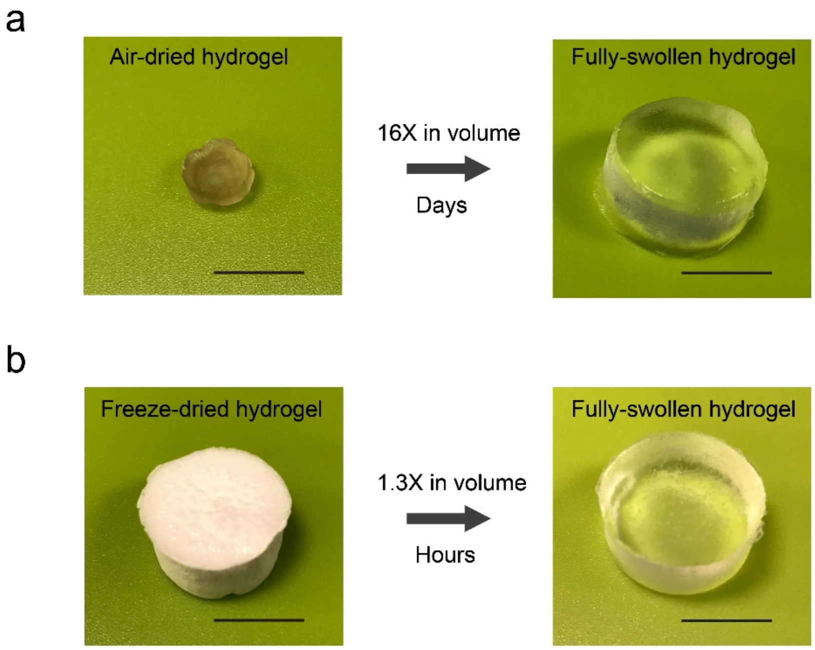

Supplementary Figure 5. Air-dried and freeze-dried polyacrylamide-agar hydrogels. (**a**) The air-dried sample without pores swells in water with high ratio and low speed. (**b**) The freeze-dried samples with pores swells in water with high speed and low ratio. Scale bars are 10 mm.

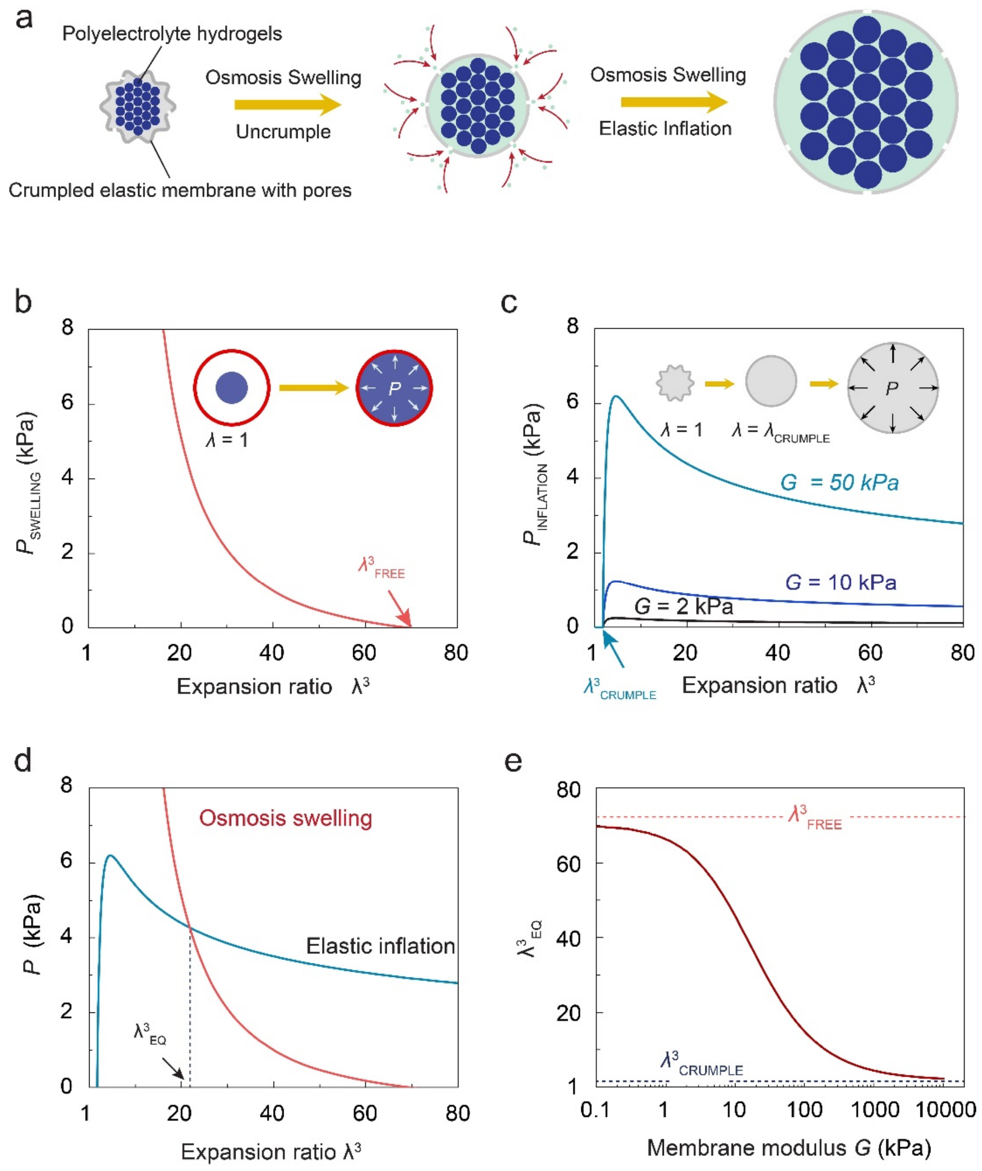

Supplementary Figure 6. Model for equilibrium swelling ratio of the ingestible hydrogel devices. (**a**) Schematic illustration of the hydrogel device expansion. (**b**) Numerical results depicting swelling-induced pressure versus constrained expansion ratio. The inset illustrates a polyelectrolyte hydrogel in reference state and constrained swollen state. (**c**) Numerical results depicting inflation pressure versus expansion ratio for a set of membranes with different moduli. The inset illustrates an elastic membrane in reference state, uncrumpled state, and inflated state. (**d**) The overlapped inflation pressure and swelling-induced pressure versus expansion ratio. The intersection of the two curves determines the swelling ratio at equilibrium state of the hydrogel devices. (**e**) Plot of expansion ratio versus membrane modulus.

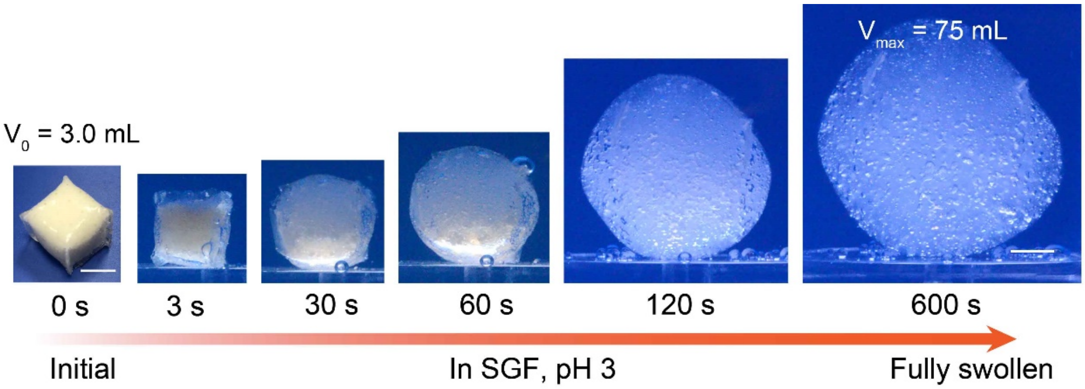

Supplementary Figure 7. High swelling speed and high swelling ratio of the ingestible hydrogel devices. Time-lapse images of the hydrogel swelling in SGF (pH 3). Scale bars are 10 mm.

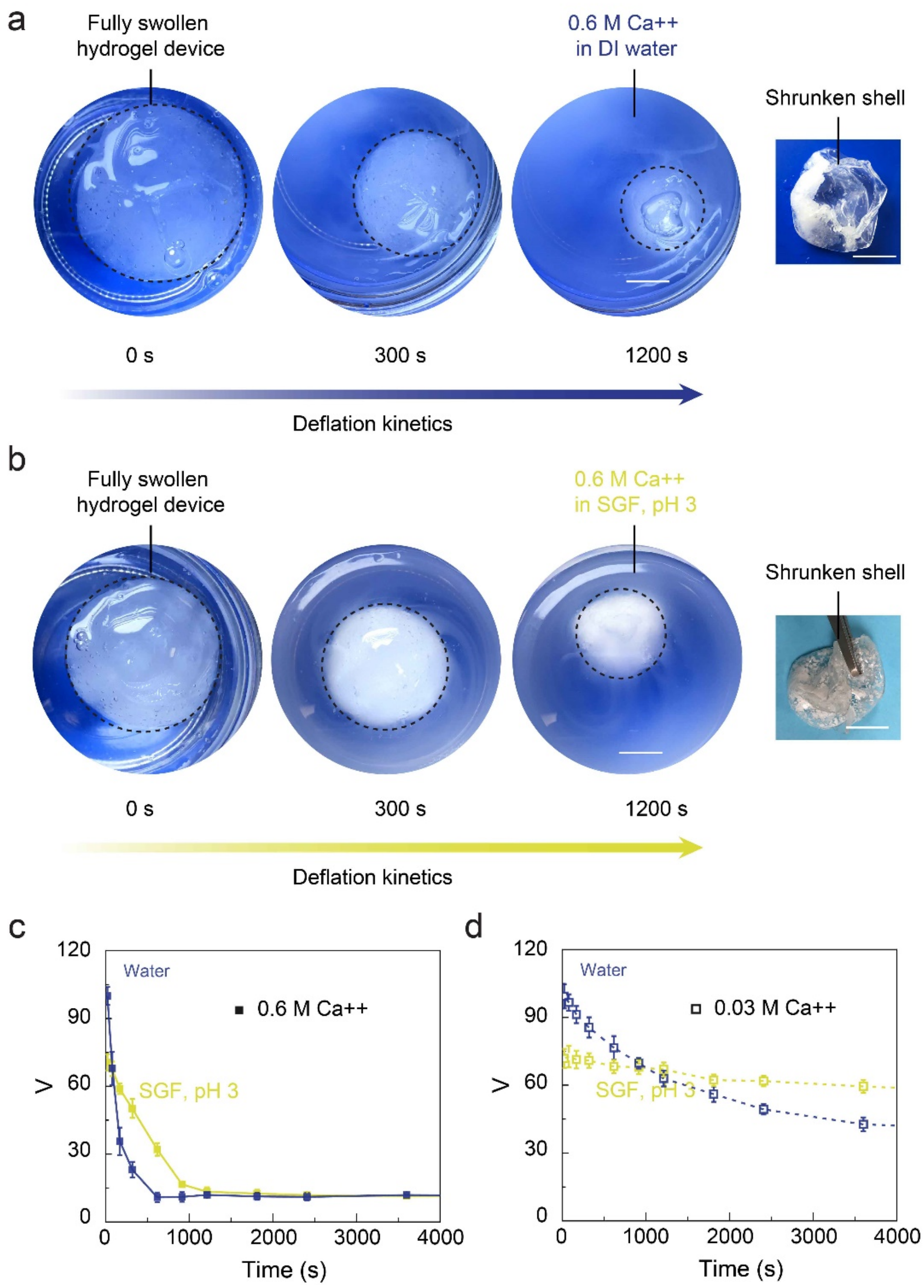

Supplementary Figure 8. Deswelling of the ingestible hydrogel devices. (**a**) Time-lapse images of the $CaCl_2$ (0.6 M) induced deswelling of the hydrogel device in water (pH 7). (**b**) Time-lapse images of the $CaCl_2$ (0.6 M) induced deswelling of the hydrogel device in SGF (pH 3). (**c**) Volumetric deflation kinetics of the hydrogel device induced by $CaCl_2$ (0.6 M) in different media. (**d**) Volumetric deflation kinetics of the hydrogel device induced by $CaCl_2$ (0.03 M) in different media. Values in **c** and **d** represent the mean ± s.d. (N = 3). Scale bars are 10 mm in **a** and **b**.

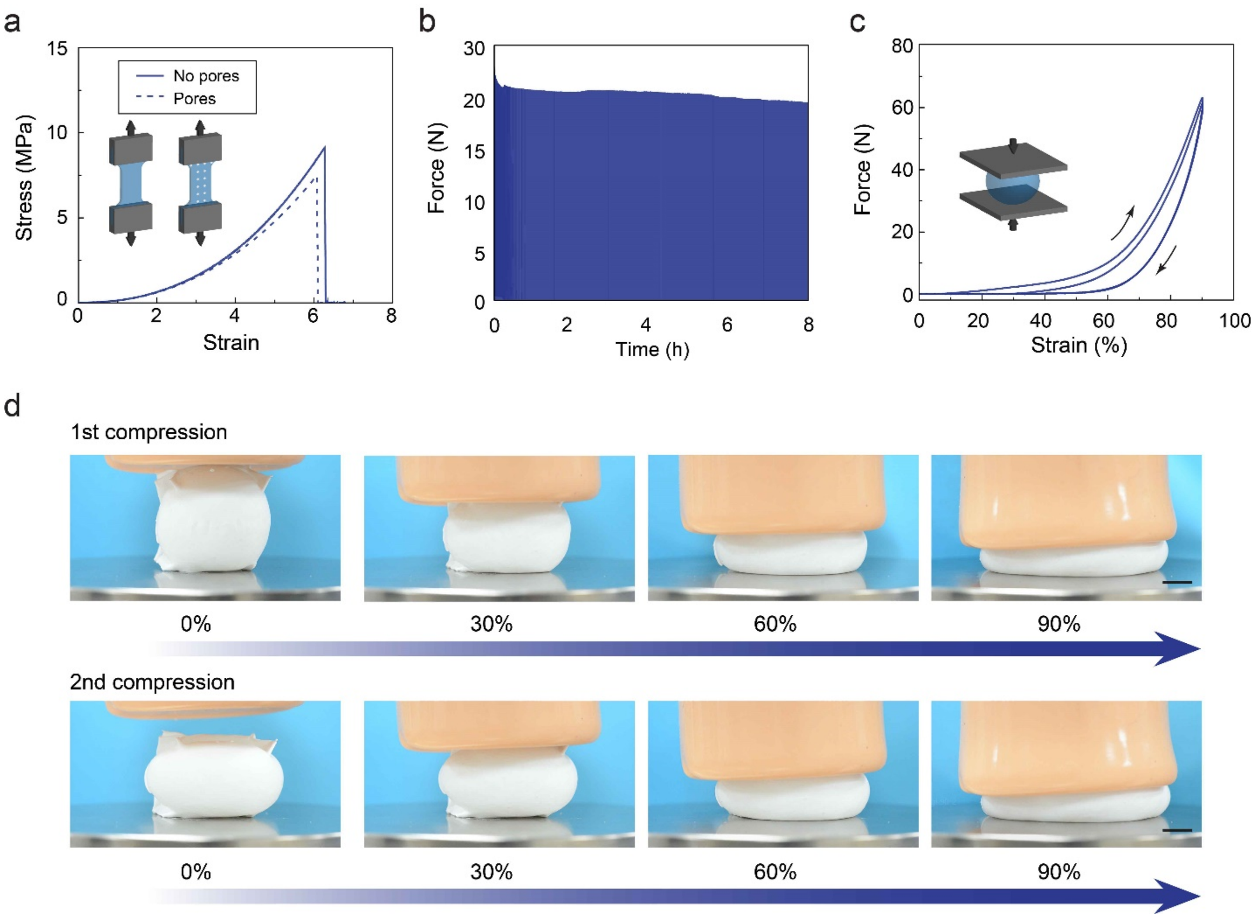

Supplementary Figure 9. Mechanical robustness of the ingestible hydrogel device in water. (**a**) True stress-stretch curves of the PVA hydrogel membranes with and without pores, which have been pre-immersed in water at 37°C for 12 h. (**b**) Measured compressive forces applied to a hydrogel device (diameter ~ 4.8 cm at undeformed state), which is kept in water under 1,920 cycles of compressive strain for 8 h on day 14. (**c**) Force-strain curves of a water-saturated hydrogel device (diameter ~ 3.6 cm at undeformed state) exposed to a maximum compressive force of 63 N and a strain of 90% for two cycles. (**d**) Time-lapse images of the water-saturated hydrogel device exposed to a maximum compressive force of 63 N and a strain of 90%. N = 3 for each test. Scale bars are 10 mm in **d**.

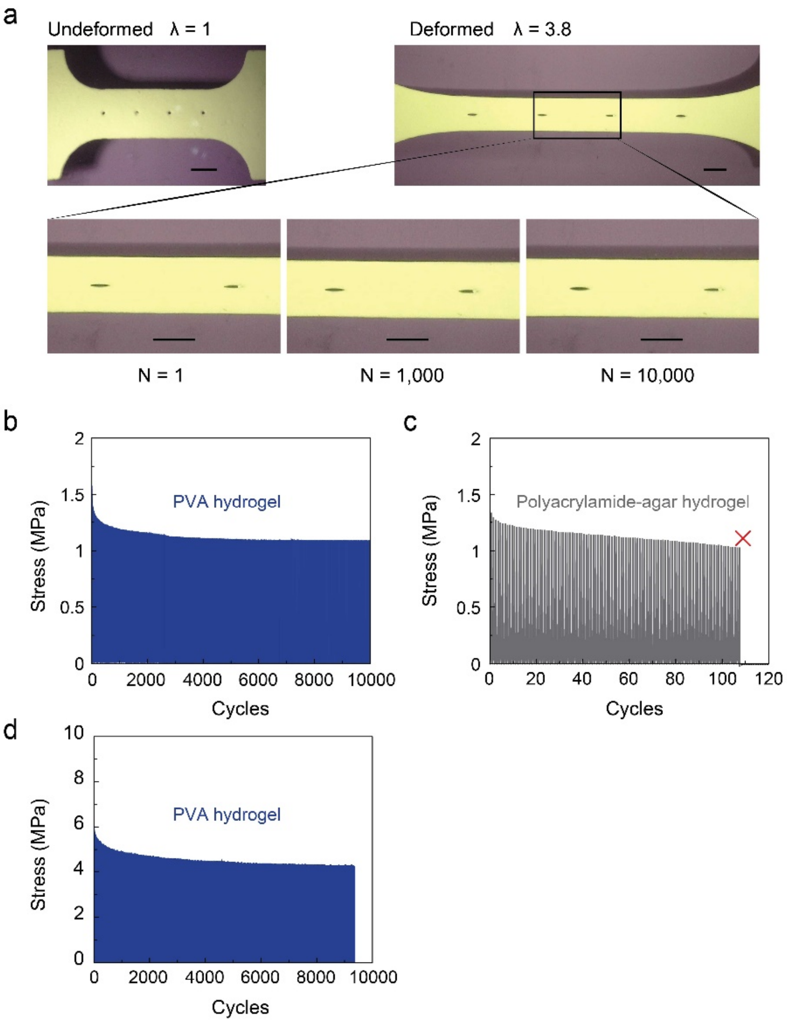

Supplementary Figure 10. Comparison of long-term strength under cyclic tensile test between two hydrogel membranes. (**a**) The undeformed state (left) and the deformed state (right) of the porous antifatigue PVA hydrogel membrane. The sample remains stable after 1, 1000, and 10,000 cycles of tensile loading. (**b**) The PVA hydrogel membrane can sustain true stress of at least 1.2 MPa for 10,000 cycles of tensile loading. (**c**) The polyacrylamide-agar hydrogel can reach the maximum true stress of 1.2 MPa but ruptures within 110 cycles of tensile loading. (**d**) The PVA hydrogel membrane can sustain true stress of 4.3 MPa for 9,000 cycles of tensile loading. N = 3 for each test. The strain rates are set as 5 mm s^{-1}. N = 3 for each test. Scale bars are 5 mm in **a**.

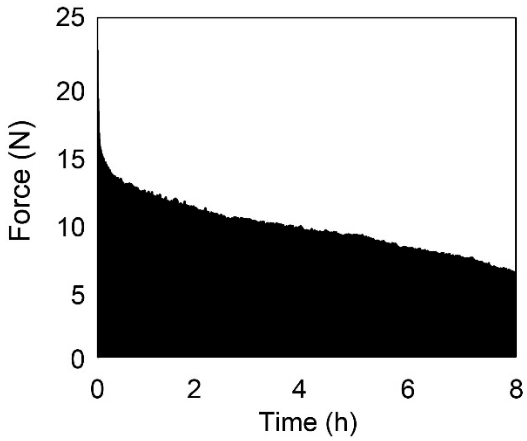

Supplementary Figure 11. Fatigue performance of the ingestible hydrogel device comprising the alternative polyacrylamide-agar hydrogel membrane. Measured cyclic compressive forces applied to the hydrogel device, which is immersed in SGF (pH 3) under 1,920 cycles of 40% compressive strain at a strain rate of 2 mm s^{-1} for 8 h on day 1. N = 3 for the test.

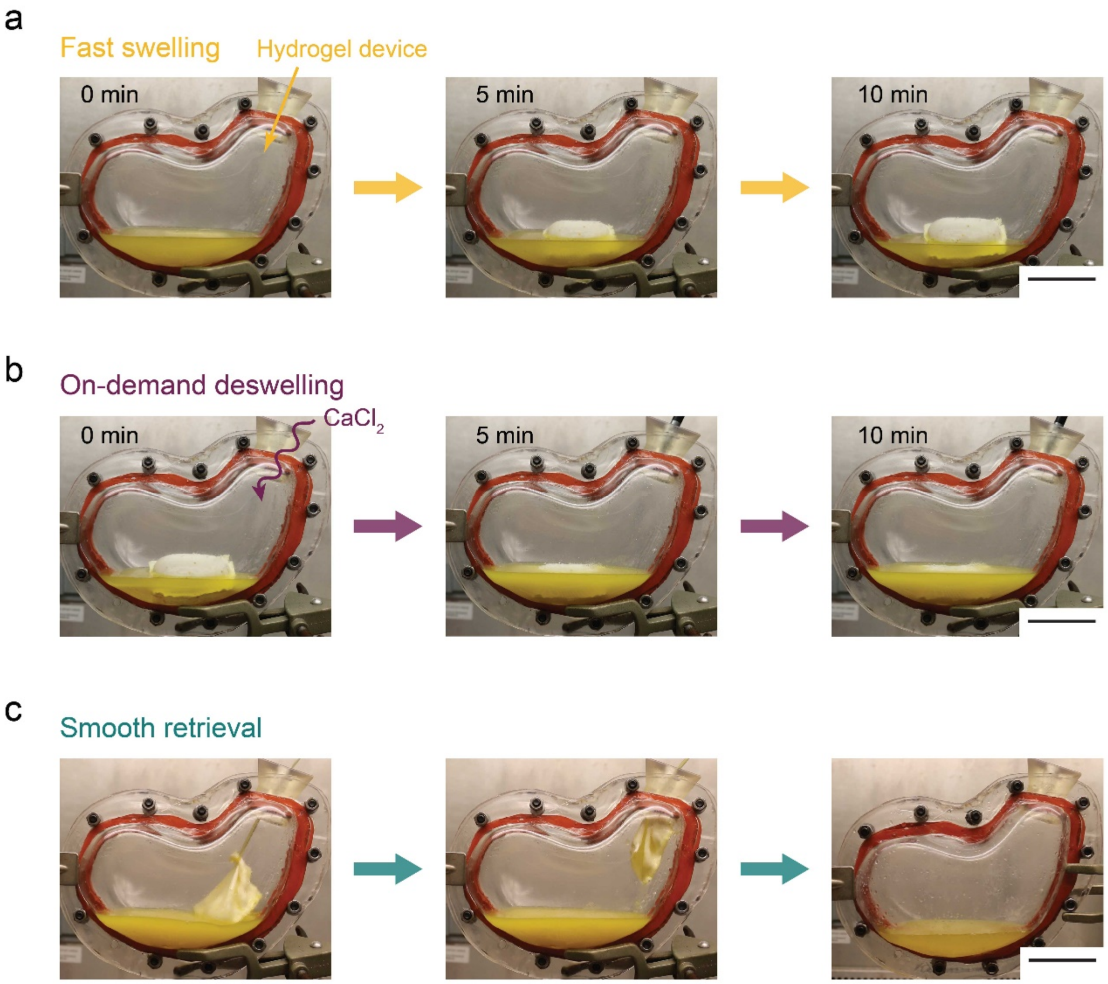

Supplementary Figure 12. Ex vivo performance of the ingestible hydrogel devices in a plastic stomach model containing porcine gastric fluid. Time-lapse images of swelling (**a**), calcium chloride triggered deswelling (**b**), and retrieval of the hydrogel devices (**c**). Scale bars are 5 cm.

Supplementary Table 1. Summary of the *in vivo* gastric residence time of ingestible hydrogel devices in a porcine model.

Hydrogel device		Animal weight on day 0 (kg)	Time (day)
Swellable	S1	39	9
	S2	42	13
	S3	38	29
Non-swellable	N1	32	3
	N2	41	6
	N3	48	6

Supplementary Table 2. Comparison of mechanical and swelling performances of existing hydrogels for gastric retention. The crosses represent the Achilles' heel of each hydrogel.

**Hydrogels**	**Mechanical properties**		**Swelling properties**		**In vivo test**	
	**Young's modulus**	**Strength of swollen gel in SGF**	**Swelling ratio in volume**	**Swelling time**	**Gastric retention time**	**Animal model**
Superporous hydrogels[5,6]	< 10 kPa	29 kPa ×	3.8-90	6 min	4-32 h	Dog
Stretchable superabsorbent hydrogels[7]	1 kPa	Not available	1000	> 1 day ×	Not available	Not available
Triggerable tough hydrogels[8]	75 kPa	Not available	2.7 ×	6 days ×	7-9 days	Pig
pH responsive hydrogels[9]	7.24 MPa	~ 0 ×	10	1 day ×	2 day	Rabbit
Ingestible, gastric-retentive hydrogel devices (our study)	3-10 kPa	70 kPa	20-100	10 min	9-29 days	Pig

Supplementary References

1. Verron, E. & Marckmann, G. Numerical analysis of rubber balloons. *Thin-walled Structures* **41**, 731-746 (2003).
2. Hong, W., Zhao, X. & Suo, Z. Large deformation and electrochemistry of polyelectrolyte gels. *Journal of the Mechanics and Physics of Solids* **58**, 558-577 (2010).
3. Hong, W., Zhao, X., Zhou, J. & Suo, Z. A theory of coupled diffusion and large deformation in polymeric gels. *Journal of the Mechanics and Physics of Solids* **56**, 1779-1793 (2008).
4. Zhao, X. A theory for large deformation and damage of interpenetrating polymer networks. *Journal of the Mechanics and Physics of Solids* **60**, 319-332 (2012).
5. Chen, J., Blevins, W.E., Park, H. & Park, K. Gastric retention properties of superporous hydrogel composites. *Journal of Controlled Release* **64**, 39-51 (2000).
6. Chen, J., Park, H. & Park, K. Synthesis of superporous hydrogels: hydrogels with fast swelling and superabsorbent properties. *Journal of Biomedical Materials Research* **44**, 53-62 (1999).
7. Cipriano, B.H.*, et al.* Superabsorbent hydrogels that are robust and highly stretchable. *Macromolecules* **47**, 4445-4452 (2014).
8. Liu, J.*, et al.* Triggerable tough hydrogels for gastric resident dosage forms. *Nature Communications* **8**, 124 (2017).
9. Wu, T.*, et al.* A pH-responsive biodegradable high-strength hydrogel as potential gastric resident filler. *Macromolecular Materials and Engineering* **303**, 1800290 (2018).
