# Supplementary material for: Ingestible hydrogel device
Source: Nat Commun. 2019 Jan 30;10:493. doi: 10.1038/s41467-019-08355-2 (PMC6353937; doi:10.1038/s41467-019-08355-2)
Supplement: Supplementary file 3 — Description of Additional Supplementary Files [file 41467_2019_8355_MOESM3_ESM.pdf]

Description of Additional Supplementary Files

File Name: Supplementary Movie 1
Description: Ingestible hydrogel device swelling in water. The movie plays at 32X realtime speed.

File Name: Supplementary Movie 2
Description: Ingestible hydrogel device swelling in simulated gastric fluid (SGF, pH 3). The movie plays at 32X real-time speed.

File Name: Supplementary Movie 3
Description: Ingestible hydrogel device deswelling in water induced by 0.6 M $CaCl_2$. The movie plays at 40X real-time speed.

File Name: Supplementary Movie 4
Description: Ingestible hydrogel device deswelling in SGF induced by 0.6 M $CaCl_2$. The movie plays at 40X real-time speed.

File Name: Supplementary Movie 5
Description: Compression of water-saturated ingestible hydrogel device to 90% strain. The movie plays at 4X real-time speed. The white-colored hydrogel device contains barium sulfate in the membrane.

File Name: Supplementary Movie 6
Description: Compression of SGF-saturated ingestible hydrogel device to 90% strain. The movie plays at 4X real-time speed. The white-colored hydrogel device contains barium sulfate in the membrane.
